# Intra-Abdominal Actinomycosis: An Indolent Masquerader of Malignancy

**DOI:** 10.7759/cureus.50215

**Published:** 2023-12-09

**Authors:** Catherine McKeever, Balaji Jayasankar, Nithish Mathew Simon, Yasser Abdul Aal, Andreas Papadopoulos

**Affiliations:** 1 General Surgery, North West Anglia NHS Foundation Trust, Huntingdon, GBR; 2 Surgery, The Belfast Health and Social Care Trust, Belfast, GBR; 3 Surgery, Maidstone and Tunbridge Wells NHS Trust, Tunbridge Wells, GBR; 4 Gynecology, Maidstone and Tunbridge Wells NHS Trust, Tunbridge Wells, GBR

**Keywords:** constitutional symptoms, actinomyces israelii, dermoid ovarian cyst, abdominal actinomycosis, multiorgan actinomycosis

## Abstract

This report describes the case of a 54-year-old female who presented with the constitutional symptoms of lethargy, weight loss, and asthenia. She had been extensively investigated for possible gynaecological malignancy but with no definitive outcome achieved. The symptoms were persistent and, partly due to occurring during the coronavirus disease 2019 (COVID-19) pandemic, a decision was made to progress with surgical management. Following an oncology multidisciplinary meeting, a decision was made for a total abdominal hysterectomy and bilateral salpingo-oophorectomy. Intra-operatively, there was an incidental finding of an extensive tumour infiltrating the liver, colon, anterior abdominal wall and urinary bladder. A surgical resection with ileostomy was performed on suspicion of an underlying malignancy. Unexpectedly, the histopathological diagnosis revealed actinomycosis. Following this discovery, our entire management plan was altered, and the patient was treated with a prolonged course of antibiotics and recovered well.

## Introduction

Actinomycosis is an uncommon indolent infection predominantly caused by the bacterium *Actinomyces Israeli *in humans [1,2]. Intra-abdominal actinomycosis is a chronic granulomatous infection that presents as a slow infiltrative mass spreading into adjacent tissues [3]. Abdominal actinomycosis masquerading as an intra-abdominal tumour is rare, and diagnosing abdominal actinomycosis is often not straightforward [1,3]. Such reports have been infrequent; however, this poses an interesting problem as the treatment modality changes completely [1-3]. We discuss a case where actinomycosis mimicked an intra-abdominal tumour and presented interesting diagnostic challenges.

## Case presentation

A 54-year-old female, who worked as a manager in a bar and lived with her partner and her daughter, presented to a district general hospital in the United Kingdom in early 2020, during the peak of the coronavirus disease 2019 (COVID-19) pandemic, with a few months’ history of fatigue, weight loss, and shortness of breath on exertion. She was an ex-smoker with a 20-pack-year history and had a background of recurrent chest infections. A CT of the thorax and abdomen revealed bronchiectasis in the lungs and a right-sided dermoid cyst. She was treated for her pneumonia and commenced on an inhaler for chronic obstructive pulmonary disease (COPD). She was also subsequently found to be severely anaemic and thus received a blood transfusion, followed by iron supplementation. Of potential note in her past medical history, she had also previously undergone sterilisation and copper intra-uterine contraceptive device insertion. There was no family history of malignancy or chronic disease.

Investigations 

**Figure 1:**
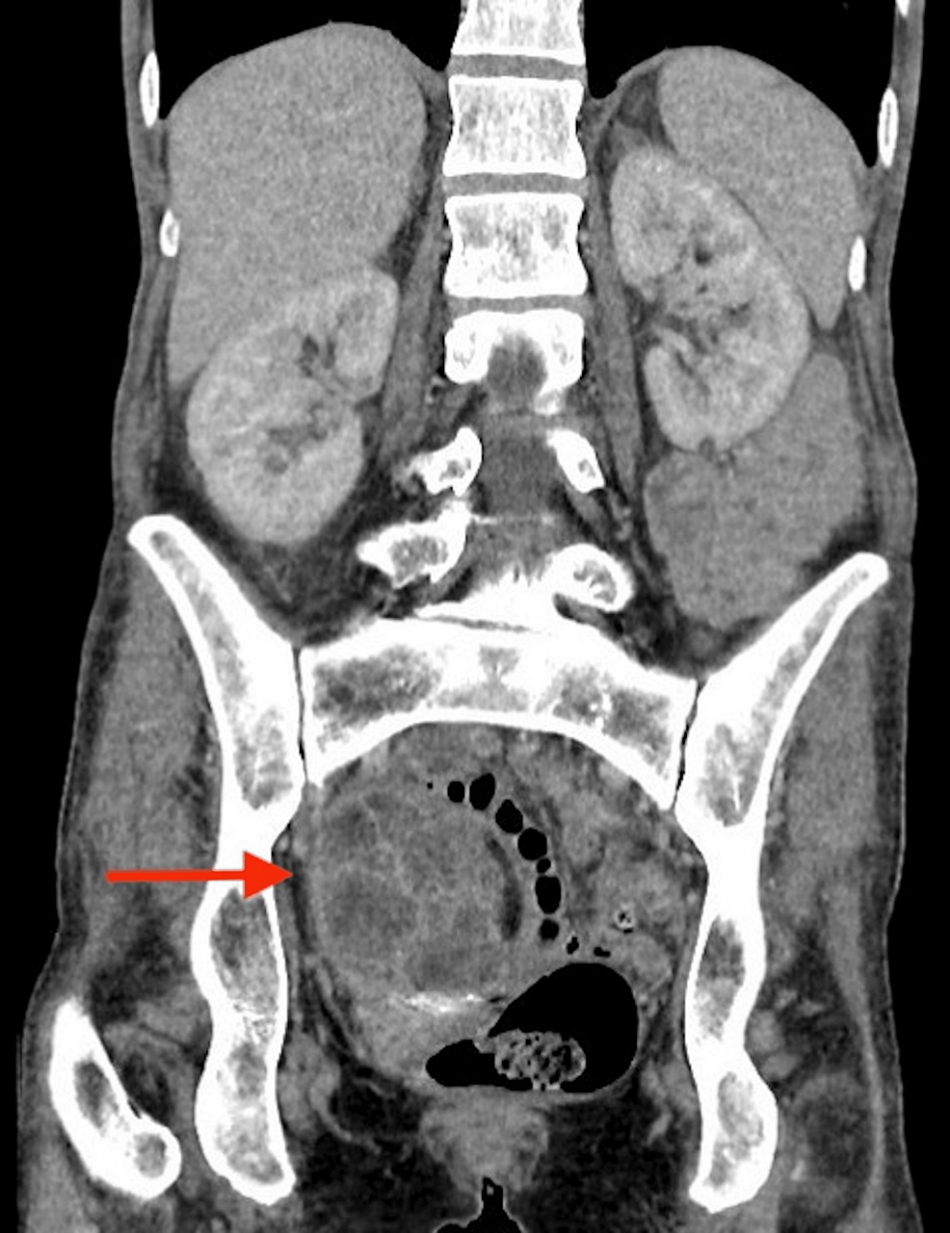
Coronal section image of CT abdomen demonstrating right adnexal mass Mixed density right adnexal mass inseparable from right ovarian dermoid and the uterus, abutting the bowel.

As the patient’s symptoms of fatigue and significant weight loss persisted following initial treatment, she was evaluated again with a CT of the thorax and abdomen. In addition to the previously detected dermoid cyst, this time the imaging also revealed a complex right adnexal mass measuring 6.3x4.9x6 cm (Figure 1).

**Figure 2:**
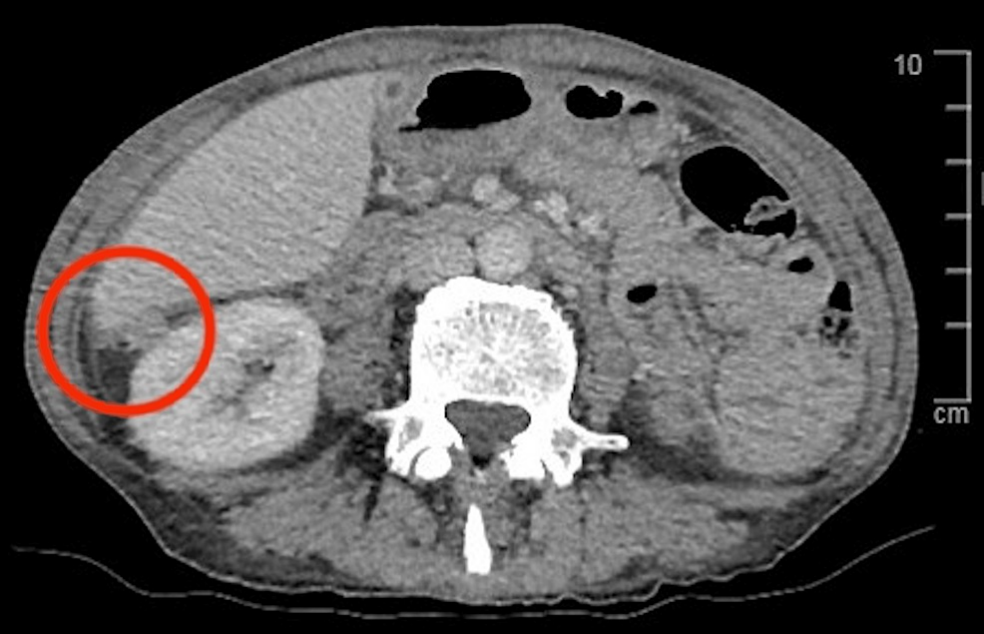
CT demonstrating multi-focal enhancing soft tissue densities in the peritoneum Soft tissue density nodule is present close to the liver, measuring 15 mm.

There were also sub-centimetric external iliac and para-aortic lymph nodes noted, along with omental nodules. There were multi-focal enhancing soft tissue densities in the peritoneum which were suggestive of a malignant aetiology (Figure 2).

Blood investigations revealed an elevated white cell count (27.3x10^9^/L), which had been raised since her treatment for pneumonia. Tumour markers were also requested, which were reported as non-specific high-normal levels. These included (normal values in brackets): CA19-9 of 50 kU/L (<27); CA125 of 20 U/mL (<35); carcinoembryonic antigen (CEA) of 4 ug/L (<4); C-reactive protein (CRP) of 111 mg/L (<5); and erythrocyte sedimentation rate (ESR) of 124 mm/hour (<29). 

The patient was accepted under the gynaecology-oncology team and following an oncology multi-disciplinary team meeting, she underwent two tissue biopsies from the omental nodule and the peritoneum. These were found to be negative for malignancy, so a subsequent tissue biopsy of a nodule around the umbilicus was performed, failing to reveal malignant tissue. A positron emission tomography (PET) scan also provided no further elucidation. 

Differential diagnosis 

The patient’s symptoms persisted, and the clinical decisions surrounding whether to operate were complicated by this occurring during the COVID-19 period. Our primary concern was potential gynaecological malignancy. However, other differential diagnoses of consideration were mesenteric panniculitis and retroperitoneal fibrosis. In addition, the presence of plasma cells on the biopsies prompted us to evaluate for IgG4 disease. Ultimately, given her clinical state, she consented to a laparotomy and proceeded.

Treatment 

She underwent a total abdominal hysterectomy with bilateral salpingo-oophorectomy under the gynaecology team, and the general surgical team’s input was sought for intra-operative incidental findings. Intra-operatively, there was a 4x4x5 cm transverse colonic mass and a corresponding 6x6x5 cm mass at the gallbladder fossa that was infiltrating the gastric antrum and segment IV of the left hepatic lobe of the liver. In addition, there was a terminal ileal mass and a 10x8x6 cm mass on the left lower anterior abdominal wall infiltrating the bladder. 

Due to these findings, she underwent ante-colic gastrojejunostomy and resection of terminal ileal mass with ileostomy formation. This was followed by resection of the anterior abdominal wall mass with partial cystectomy with clearance of left iliac lymph nodes and reconstruction of the anterior abdominal wall using a mesh. Her immediate postoperative recovery was unremarkable. However, she did have difficulties with managing her stoma and diet in the weeks following the operation. 

Outcome and follow-up 

**Figure 3:**
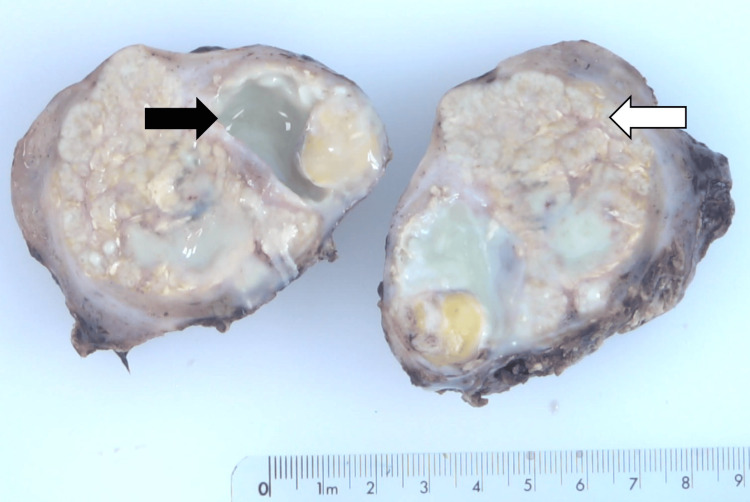
Gross appearance of bisected right ovary with features of dermoid cyst with abscesses Black arrow is abscess cavity with adjacent yellow nodule of adipose tissue of the dermoid. White arrow represents inflamed ovarian parenchyma.

The histopathology of the ovaries revealed features suggestive of dermoid cyst with abscesses (Figure 3).

**Figure 4:**
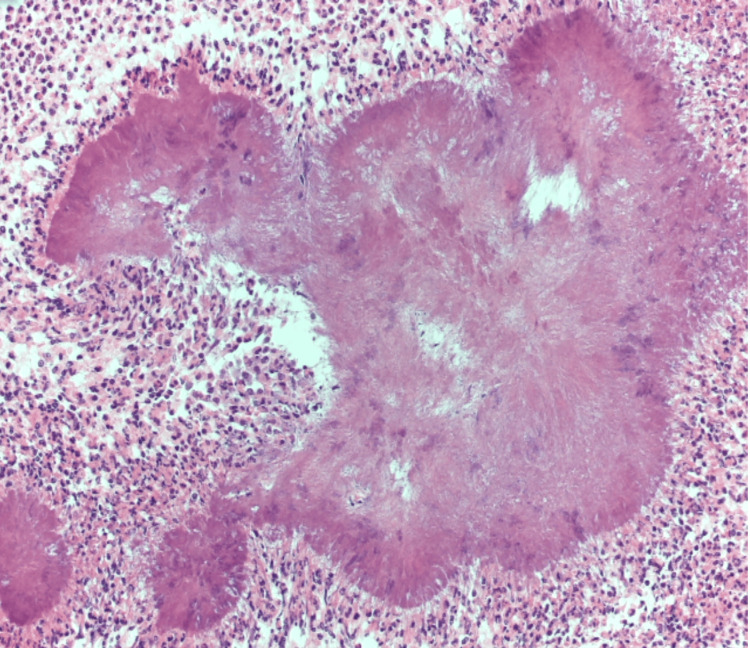
Ovarian abscesses (H&E stain; x200) Colonies of actinomycosis surrounded by purulent exudates

The abscesses revealed colonies of actinomycosis which had spread and mimicked malignancy (Figure 4).

Following discussion with and review by the microbiology team, she was started on amoxicillin one gram three times a day for six months and a possible one-year extension following review. In addition, a follow-up with the Infectious Diseases team was organised. After the surgery, the patient was commenced on total parenteral nutrition for two weeks, with a gradual reintroduction of soft diet. Postoperative recovery was uneventful with a gradual build-up of physical strength and gaining confidence in stoma management. Encouragingly, at the time of discharge, she was independent with the management of her stoma. On subsequent follow-up, she was clinically well, had gained weight, and was being considered for a stoma reversal.

## Discussion

Intra-abdominal actinomycosis presents as a granulomatous mass with localised abscesses and pus [1,2]. Often this further leads to necrosis and reactive fibrosis [2-4]. The incidence has been reported to be 1:300,000 with a male preponderance of 3:1 and usually affects individuals between second and sixth decade [1,5]. 

The causative bacterium *Actinomyces israelii* is a gram-positive, filamentous, anaerobic bacterium which usually resides in the oral cavity, gastrointestinal and female genital tract [5]. The organism has an intrinsic low virulence and remains indolent, especially if the mucosal barrier is intact. It is only when the normal mucosal barrier is breached or in immunocompromised individuals that the infection becomes apparent [5,6]. This has been supported by the fact that the infection is found following foreign body insertion, localised inflammation, bowel perforation, ruptured appendix, and following intrauterine device insertion. Also of note is the lack of haematogenous or lymphatic spread. Most predominant infections have been of cervicofacial (50%), abdomen and pelvis (20%), followed by thoracic (15%) [1,6]. 

Clinical presentation of intra-abdominal actinomycosis can be similar to chronic diseases but lacks aggression. Patients usually present with weight loss, asthenia, lethargy, and mild constitutional symptoms [2,6]. These indeterminate symptoms, in addition to the rarity of the disease, are likely to lead the clinician toward considering the more common differentials of malignancy, lymphoma, tuberculosis, and/or inflammatory bowel diseases. Due to the condition's growth pattern, it is often misinterpreted leading to a delay in diagnosis and treatment [4,6,7]. 

Preoperative diagnosis is extremely difficult and the paucity of definitive findings with laboratory and radiological diagnosis does not help in this regard. Radiologically, CT scan, MRI and barium swallow have demonstrated clinical use, but only a CT scan has been considered to demonstrate a clinical benefit [2,5,7]. CT imaging can be useful in visualising the extent and the relation to adjacent structures but cannot be used as a definitive diagnostic tool. The beneficial application of CT imaging in this context can be seen in revealing focal areas of a solid mass with low attenuation, and the presence of cystic areas with thickened walls can be invaluable for guided biopsies [1,5]. 

Histological diagnosis and microbiological culture are paramount in building the diagnostic picture, as well as forming the basis for further treatment. Under the microscope, multiple gram-positive branching hyphae with or without sulphur granules are seen. Anaerobic culture of the organisms would be the gold standard; however, this is challenging in practice. As anaerobic cultures are not routinely performed on all biopsy samples, prior planning is required to obtain the appropriate anaerobic culture equipment [4,6,7]. In addition to this, obtaining adequate biopsy samples can be limited by the depth of tissue sampling achievable [8]. Furthermore, results may take up to two to four weeks to be returned [4,6,7]. 

Surgical intervention can be utilised in debulking the tumour and establishing a preoperative diagnosis is seldom achieved. Following this, antimicrobial therapy forms the basis of treatment [1,2,5]. Typically, the antibiotic is a high dose penicillin, given for a prolonged duration as the desmoplastic reaction can limit the drug penetration. The drug of choice is penicillin initially administered parenterally [8]. An alternative of tetracycline, clindamycin, and erythromycin is recommended for penicillin-allergic individuals. The recommendation has been to use intravenous penicillin G (10-20 million units) for four to six weeks, followed by oral penicillin or amoxicillin 2-4 g/day. However, there has been no clear consensus on the dose and duration of therapy and this could be modified on a case-by-case basis. The course of treatment typically ranges from six to 18 months. The long duration helps in curing as well as preventing relapse; however, the side effects could pose an issue with compliance [4,6,7]. 

## Conclusions

A learning point from this case was that the overall time to diagnosis and delay in treatment from the initial symptom onset can be highly distressing for the patient. On reflection, culturing the biopsy samples may have demonstrated the presence of the organism sooner; however, in isolation, this would not likely have changed the overall time course of definitive treatment for this patient as surgical management had been identified as the preferred management option. In managing this rare condition in this 54-year-old patient, surgery was crucial in debulking and obtaining a definitive diagnosis; however, antimicrobial therapy and supportive measures continue to be the mainstay of ongoing treatment. 

Abdominal actinomycosis could be suspected in patients with prolonged constitutional symptoms and imaging suggestive of intra-abdominal mass with no apparent cause. However, as actinomycosis infection is an uncommon condition, the medical team would have to have a high degree of suspicion to consider such an uncommon differential. Suspicion of uncommon infections such as actinomycosis may increase with immunocompromised individuals or a foreign body or intrauterine device insertion. Moving forward, compilation and review of similar cases may help to improve the medical literature available in guiding the treatment of such cases of uncommon infections.

## Appendices

Patient’s perspective

My symptoms were really worrying and I was initially disappointed that I had been treated only for my chest infection. It was extremely distressing as I had to quit my job and found it extremely difficult to manage things by myself. I had lost around 5 stones of weight in a span of six months and this made me really weak. I was worried prior to the surgery due to the diagnosis of malignancy. Following the surgery, it had been tough to cope with the stoma and diet. But I believe I am making progress. I am extremely relieved that this is not cancer and that it is an infection that can be treated. It was explained to me that this is really rare. But I am hopeful and positive about making a good recovery
